# Supplementary figures and images for: An in vitro study to assess the effect of hyaluronan-based gels on muscle-derived cells: Highlighting a new perspective in regenerative medicine
Source: PLoS One. 2020 Aug 6;15(8):e0236164. doi: 10.1371/journal.pone.0236164 (PMC7410276; doi:10.1371/journal.pone.0236164)

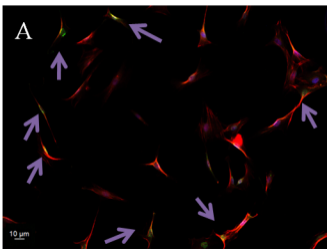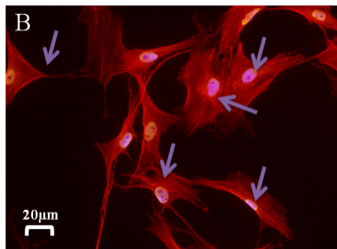

Supplement: S1 Fig — (PDF) [file pone.0236164.s001.pdf]
