# Supplementary material for: An in vitro study to assess the effect of hyaluronan-based gels on muscle-derived cells: Highlighting a new perspective in regenerative medicine
Source: PLoS One. 2020 Aug 6;15(8):e0236164. doi: 10.1371/journal.pone.0236164 (PMC7410276; doi:10.1371/journal.pone.0236164)
Supplement: S2 Fig — (DOCX) [file pone.0236164.s002.docx]

**Results**

**ANOVA**

| **ANOVA – MTT-test** | | | | | | | | | | | |
| --- | --- | --- | --- | --- | --- | --- | --- | --- | --- | --- | --- |
| **Cases** | | **Sum of Squares** | | **df** | | **Mean Square** | | **F** | | **p** | |
| V1 |  | 1049.886 |  | 4 |  | 262.472 |  | 6.139 |  | 0.009 |  |
| Residuals |  | 427.564 |  | 10 |  | 42.756 |  |  |  |  |  |
|  | | | | | | | | | | | |
| *Note.*  Type III Sum of Squares | | | | | | | | | | | |

**Post Hoc Tests**

**Standard**

| **Post Hoc Comparisons - V1** | | | | | | | | | | | | | | | |
| --- | --- | --- | --- | --- | --- | --- | --- | --- | --- | --- | --- | --- | --- | --- | --- |
|  | | | | | | **95% CI for Mean Difference** | | | |  | | | | | |
|  | |  | | **Mean Difference** | | **Lower** | | **Upper** | | **SE** | | **t** | | **p _tukey_** | |
| CTR 24h |  | H2O2 |  | 11.066 |  | -6.505 |  | 28.636 |  | 5.339 |  | 2.073 |  | 0.302 |  |
|  |  | H2O2+HCC |  | -7.371 |  | -24.942 |  | 10.200 |  | 5.339 |  | -1.381 |  | 0.652 |  |
|  |  | H2O2+HHA |  | -5.383 |  | -22.954 |  | 12.188 |  | 5.339 |  | -1.008 |  | 0.846 |  |
|  |  | H2O2+LHA |  | -13.881 |  | -31.452 |  | 3.690 |  | 5.339 |  | -2.600 |  | 0.144 |  |
| H2O2 24h |  | H2O2+HCC |  | -18.437 |  | -36.008 |  | -0.866 |  | 5.339 |  | -3.453 |  | 0.039 |  |
|  |  | H2O2+HHA |  | -16.448 |  | -34.019 |  | 1.123 |  | 5.339 |  | -3.081 |  | 0.069 |  |
|  |  | H2O2+LHA |  | -24.947 |  | -42.518 |  | -7.376 |  | 5.339 |  | -4.673 |  | 0.006 |  |
| H2O2+HCC 24h |  | H2O2+HHA |  | 1.988 |  | -15.582 |  | 19.559 |  | 5.339 |  | 0.372 |  | 0.995 |  |
|  |  | H2O2+LHA |  | -6.510 |  | -24.081 |  | 11.061 |  | 5.339 |  | -1.219 |  | 0.741 |  |
| H2O2+HHA 24h |  | H2O2+LHA |  | -8.499 |  | -26.069 |  | 9.072 |  | 5.339 |  | -1.592 |  | 0.533 |  |
|  | | | | | | | | | | | | | | | |
| *Note.*  P-value and confidence intervals adjusted for comparing a family of 5 estimates (confidence intervals corrected using the tukey method). | | | | | | | | | | | | | | | |

**Results**

**ANOVA**

| **ANOVA – MTT-test** | | | | | | | | | | | |
| --- | --- | --- | --- | --- | --- | --- | --- | --- | --- | --- | --- |
| **Cases** | | **Sum of Squares** | | **df** | | **Mean Square** | | **F** | | **p** | |
| V1 |  | 6598.043 |  | 4 |  | 1649.511 |  | 250.894 |  | < .001 |  |
| Residuals |  | 65.745 |  | 10 |  | 6.575 |  |  |  |  |  |
|  | | | | | | | | | | | |
| *Note.*  Type III Sum of Squares | | | | | | | | | | | |

**Post Hoc Tests**

**Standard**

| **Post Hoc Comparisons - V1** | | | | | | | | | | | | | | | |
| --- | --- | --- | --- | --- | --- | --- | --- | --- | --- | --- | --- | --- | --- | --- | --- |
|  | | | | | | **95% CI for Mean Difference** | | | |  | | | | | |
|  | |  | | **Mean Difference** | | **Lower** | | **Upper** | | **SE** | | **t** | | **p _tukey_** | |
| CTR 48h |  | H2O2 |  | 10.066 |  | 3.176 |  | 16.956 |  | 2.094 |  | 4.808 |  | 0.0107 |  |
|  |  | H2O2+HCC |  | -50.286 |  | -57.176 |  | -43.396 |  | 2.094 |  | -24.019 |  | < .001 |  |
|  |  | H2O2+HHA |  | -5.738 |  | -12.628 |  | 1.152 |  | 2.094 |  | -2.741 |  | 0.116 |  |
|  |  | H2O2+LHA |  | -2.512 |  | -9.402 |  | 4.378 |  | 2.094 |  | -1.200 |  | 0.752 |  |
| H2O2 48h |  | H2O2+HCC |  | -60.352 |  | -67.242 |  | -53.462 |  | 2.094 |  | -28.827 |  | < .001 |  |
|  |  | H2O2+HHA |  | -15.804 |  | -22.694 |  | -8.914 |  | 2.094 |  | -7.549 |  | < .001 |  |
|  |  | H2O2+LHA |  | -12.578 |  | -19.468 |  | -5.688 |  | 2.094 |  | -6.008 |  | < .001 |  |
| H2O2+HCC 48h |  | H2O2+HHA |  | 44.548 |  | 37.658 |  | 51.438 |  | 2.094 |  | 21.278 |  | < .001 |  |
|  |  | H2O2+LHA |  | 47.774 |  | 40.884 |  | 54.664 |  | 2.094 |  | 22.819 |  | < .001 |  |
| H2O2+HHA 48h |  | H2O2+LHA |  | 3.226 |  | -3.664 |  | 10.116 |  | 2.094 |  | 1.541 |  | 0.562 |  |
|  | | | | | | | | | | | | | | | |
| *Note.*  P-value and confidence intervals adjusted for comparing a family of 5 estimates (confidence intervals corrected using the tukey method). | | | | | | | | | | | | | | | |

**Results**

**ANOVA**

| **ANOVA – MTT-test** | | | | | | | | | | | |
| --- | --- | --- | --- | --- | --- | --- | --- | --- | --- | --- | --- |
| **Cases** | | **Sum of Squares** | | **df** | | **Mean Square** | | **F** | | **p** | |
| V1 |  | 617156.881 |  | 4 |  | 154289.220 |  | 185.994 |  | < .001 |  |
| Residuals |  | 8295.408 |  | 10 |  | 829.541 |  |  |  |  |  |
|  | | | | | | | | | | | |
| *Note.*  Type III Sum of Squares | | | | | | | | | | | |

**Post Hoc Tests**

**Standard**

| **Post Hoc Comparisons - V1** | | | | | | | | | | | | | | | |
| --- | --- | --- | --- | --- | --- | --- | --- | --- | --- | --- | --- | --- | --- | --- | --- |
|  | | | | | | **95% CI for Mean Difference** | | | |  | | | | | |
|  | |  | | **Mean Difference** | | **Lower** | | **Upper** | | **SE** | | **t** | | **p _tukey_** | |
| CTR 72h |  | H2O2 |  | 65.831 |  | -11.564 |  | 143.225 |  | 23.517 |  | 2.799 |  | 0.005 |  |
|  |  | H2O2+HCC |  | -518.741 |  | -596.136 |  | -441.346 |  | 23.517 |  | -22.059 |  | < .001 |  |
|  |  | H2O2+HHA |  | -142.968 |  | -220.363 |  | -65.573 |  | 23.517 |  | -6.079 |  | < .001 |  |
|  |  | H2O2+LHA |  | -121.110 |  | -198.505 |  | -43.715 |  | 23.517 |  | -5.150 |  | 0.003 |  |
| H2O2 72h |  | H2O2+HCC |  | -584.572 |  | -661.966 |  | -507.177 |  | 23.517 |  | -24.858 |  | < .001 |  |
|  |  | H2O2+HHA |  | -208.799 |  | -286.193 |  | -131.404 |  | 23.517 |  | -8.879 |  | < .001 |  |
|  |  | H2O2+LHA |  | -186.940 |  | -264.335 |  | -109.546 |  | 23.517 |  | -7.949 |  | < .001 |  |
| H2O2+HCC 72h |  | H2O2+HHA |  | 375.773 |  | 298.378 |  | 453.168 |  | 23.517 |  | 15.979 |  | < .001 |  |
|  |  | H2O2+LHA |  | 397.631 |  | 320.236 |  | 475.026 |  | 23.517 |  | 16.909 |  | < .001 |  |
| H2O2+HHA 72h |  | H2O2+LHA |  | 21.858 |  | -55.537 |  | 99.253 |  | 23.517 |  | 0.929 |  | 0.879 |  |
|  | | | | | | | | | | | | | | | |
| *Note.*  P-value and confidence intervals adjusted for comparing a family of 5 estimates (confidence intervals corrected using the tukey method). | | | | | | | | | | | | | | | |

**Results**

**ANOVA**

| **ANOVA – MTT-test** | | | | | | | | | | | |
| --- | --- | --- | --- | --- | --- | --- | --- | --- | --- | --- | --- |
| **Cases** | | **Sum of Squares** | | **df** | | **Mean Square** | | **F** | | **p** | |
| V1 |  | 4854.222 |  | 4 |  | 1213.556 |  | 27.687 |  | < .001 |  |
| Residuals |  | 438.318 |  | 10 |  | 43.832 |  |  |  |  |  |
|  | | | | | | | | | | | |
| *Note.*  Type III Sum of Squares | | | | | | | | | | | |

**Post Hoc Tests**

**Standard**

| **Post Hoc Comparisons - V1** | | | | | | | | | | | | | | | |
| --- | --- | --- | --- | --- | --- | --- | --- | --- | --- | --- | --- | --- | --- | --- | --- |
|  | | | | | | **95% CI for Mean Difference** | | | |  | | | | | |
|  | |  | | **Mean Difference** | | **Lower** | | **Upper** | | **SE** | | **t** | | **p _tukey_** | |
| CTR |  | H2O2 24h |  | 16.645 |  | -1.145 |  | 34.436 |  | 5.406 |  | 3.079 |  | 0.069 |  |
|  |  | HCC24h |  | -36.392 |  | -54.183 |  | -18.602 |  | 5.406 |  | -6.732 |  | < .001 |  |
|  |  | HHA 24h |  | -15.996 |  | -33.786 |  | 1.795 |  | 5.406 |  | -2.959 |  | 0.084 |  |
|  |  | LHA24h |  | -18.944 |  | -36.735 |  | -1.154 |  | 5.406 |  | -3.505 |  | 0.036 |  |
| H2O2 24h |  | HCC24h |  | -53.037 |  | -70.828 |  | -35.247 |  | 5.406 |  | -9.811 |  | < .001 |  |
|  |  | HHA 24h |  | -32.641 |  | -50.432 |  | -14.851 |  | 5.406 |  | -6.038 |  | < .001 |  |
|  |  | LHA24h |  | -35.590 |  | -53.380 |  | -17.799 |  | 5.406 |  | -6.584 |  | < .001 |  |
| HCC24h |  | HHA 24h |  | 20.396 |  | 2.606 |  | 38.187 |  | 5.406 |  | 3.773 |  | 0.024 |  |
|  |  | LHA24h |  | 17.448 |  | -0.343 |  | 35.238 |  | 5.406 |  | 3.228 |  | 0.055 |  |
| HHA 24h |  | LHA24h |  | -2.949 |  | -20.739 |  | 14.842 |  | 5.406 |  | -0.545 |  | 0.980 |  |
|  | | | | | | | | | | | | | | | |
| *Note.*  P-value and confidence intervals adjusted for comparing a family of 5 estimates (confidence intervals corrected using the tukey method). | | | | | | | | | | | | | | | |

**ANOVA**

| **ANOVA – MTT-test** | | | | | | | | | | | |
| --- | --- | --- | --- | --- | --- | --- | --- | --- | --- | --- | --- |
| **Cases** | | **Sum of Squares** | | **df** | | **Mean Square** | | **F** | | **p** | |
| V1 |  | 4854.222 |  | 4 |  | 1213.556 |  | 27.687 |  | < .001 |  |
| Residuals |  | 438.318 |  | 10 |  | 43.832 |  |  |  |  |  |
|  | | | | | | | | | | | |
| *Note.*  Type III Sum of Squares | | | | | | | | | | | |

**Post Hoc Tests**

**Standard**

| **Post Hoc Comparisons - V1** | | | | | | | | | | | | | | | |
| --- | --- | --- | --- | --- | --- | --- | --- | --- | --- | --- | --- | --- | --- | --- | --- |
|  | | | | | | **95% CI for Mean Difference** | | | |  | | | | | |
|  | |  | | **Mean Difference** | | **Lower** | | **Upper** | | **SE** | | **t** | | **p _tukey_** | |
| CTR |  | H2O2 24h |  | 16.645 |  | -1.145 |  | 34.436 |  | 5.406 |  | 3.079 |  | 0.069 |  |
|  |  | HCC24h |  | -36.392 |  | -54.183 |  | -18.602 |  | 5.406 |  | -6.732 |  | < .001 |  |
|  |  | HHA 24h |  | -15.996 |  | -33.786 |  | 1.795 |  | 5.406 |  | -2.959 |  | 0.084 |  |
|  |  | LHA24h |  | -18.944 |  | -36.735 |  | -1.154 |  | 5.406 |  | -3.505 |  | 0.036 |  |
| H2O2 24h |  | HCC24h |  | -53.037 |  | -70.828 |  | -35.247 |  | 5.406 |  | -9.811 |  | < .001 |  |
|  |  | HHA 24h |  | -32.641 |  | -50.432 |  | -14.851 |  | 5.406 |  | -6.038 |  | < .001 |  |
|  |  | LHA24h |  | -35.590 |  | -53.380 |  | -17.799 |  | 5.406 |  | -6.584 |  | < .001 |  |
| HCC24h |  | HHA 24h |  | 20.396 |  | 2.606 |  | 38.187 |  | 5.406 |  | 3.773 |  | 0.024 |  |
|  |  | LHA24h |  | 17.448 |  | -0.343 |  | 35.238 |  | 5.406 |  | 3.228 |  | 0.055 |  |
| HHA 24h |  | LHA24h |  | -2.949 |  | -20.739 |  | 14.842 |  | 5.406 |  | -0.545 |  | 0.980 |  |
|  | | | | | | | | | | | | | | | |
| *Note.*  P-value and confidence intervals adjusted for comparing a family of 5 estimates (confidence intervals corrected using the tukey method). | | | | | | | | | | | | | | | |

**Results**

**ANOVA**

| **ANOVA - MTT-test** | | | | | | | | | | | |
| --- | --- | --- | --- | --- | --- | --- | --- | --- | --- | --- | --- |
| **Cases** | | **Sum of Squares** | | **df** | | **Mean Square** | | **F** | | **p** | |
| V1 |  | 19540.608 |  | 4 |  | 4885.152 |  | 99.516 |  | < .001 |  |
| Residuals |  | 490.891 |  | 10 |  | 49.089 |  |  |  |  |  |
|  | | | | | | | | | | | |
| *Note.*  Type III Sum of Squares | | | | | | | | | | | |

**Post Hoc Tests**

**Standard**

| **Post Hoc Comparisons - V1** | | | | | | | | | | | | | | | |
| --- | --- | --- | --- | --- | --- | --- | --- | --- | --- | --- | --- | --- | --- | --- | --- |
|  | | | | | | **95% CI for Mean Difference** | | | |  | | | | | |
|  | |  | | **Mean Difference** | | **Lower** | | **Upper** | | **SE** | | **t** | | **p _tukey_** | |
| CTR 48h |  | H2O2 48h |  | 32.375 |  | 13.548 |  | 51.202 |  | 5.721 |  | 5.659 |  | 0.002 |  |
|  |  | HCC 48h |  | -61.235 |  | -80.062 |  | -42.408 |  | 5.721 |  | -10.704 |  | < .001 |  |
|  |  | HHA 48h |  | -60.185 |  | -79.012 |  | -41.358 |  | 5.721 |  | -10.521 |  | < .001 |  |
|  |  | LHA 48h |  | -31.988 |  | -50.815 |  | -13.161 |  | 5.721 |  | -5.592 |  | 0.002 |  |
| H2O2 48h |  | HCC 48h |  | -93.610 |  | -112.437 |  | -74.783 |  | 5.721 |  | -16.363 |  | < .001 |  |
|  |  | HHA 48h |  | -92.560 |  | -111.387 |  | -73.733 |  | 5.721 |  | -16.180 |  | < .001 |  |
|  |  | LHA 48h |  | -64.363 |  | -83.190 |  | -45.536 |  | 5.721 |  | -11.251 |  | < .001 |  |
| HCC 48h |  | HHA 48h |  | 1.050 |  | -17.777 |  | 19.877 |  | 5.721 |  | 0.184 |  | 1.000 |  |
|  |  | LHA 48h |  | 29.247 |  | 10.420 |  | 48.075 |  | 5.721 |  | 5.113 |  | 0.003 |  |
| HHA 48h |  | LHA 48h |  | 28.197 |  | 9.370 |  | 47.024 |  | 5.721 |  | 4.929 |  | 0.004 |  |
|  | | | | | | | | | | | | | | | |
| *Note.*  P-value and confidence intervals adjusted for comparing a family of 5 estimates (confidence intervals corrected using the tukey method). | | | | | | | | | | | | | | | |

**Results**

**ANOVA**

| **ANOVA - MTT-test** | | | | | | | | | | | |
| --- | --- | --- | --- | --- | --- | --- | --- | --- | --- | --- | --- |
| **Cases** | | **Sum of Squares** | | **df** | | **Mean Square** | | **F** | | **p** | |
| V1 |  | 94718.417 |  | 4 |  | 23679.604 |  | 131.809 |  | < .001 |  |
| Residuals |  | 1796.513 |  | 10 |  | 179.651 |  |  |  |  |  |
|  | | | | | | | | | | | |
| *Note.*  Type III Sum of Squares | | | | | | | | | | | |

**Post Hoc Tests**

**Standard**

| **Post Hoc Comparisons - V1** | | | | | | | | | | | | | | | |
| --- | --- | --- | --- | --- | --- | --- | --- | --- | --- | --- | --- | --- | --- | --- | --- |
|  | | | | | | **95% CI for Mean Difference** | | | |  | | | | | |
|  | |  | | **Mean Difference** | | **Lower** | | **Upper** | | **SE** | | **t** | | **p _tukey_** | |
| CTR 72h |  | H2O2 72h |  | 64.663 |  | 28.646 |  | 100.680 |  | 10.944 |  | 5.909 |  | 0.001 |  |
|  |  | HCC 72h |  | -150.740 |  | -186.758 |  | -114.723 |  | 10.944 |  | -13.774 |  | < .001 |  |
|  |  | HHA 72h |  | -62.580 |  | -98.597 |  | -26.563 |  | 10.944 |  | -5.718 |  | 0.001 |  |
|  |  | LHA 72h |  | -125.746 |  | -161.763 |  | -89.729 |  | 10.944 |  | -11.490 |  | < .001 |  |
| H2O2 72h |  | HCC 72h |  | -215.403 |  | -251.420 |  | -179.386 |  | 10.944 |  | -19.683 |  | < .001 |  |
|  |  | HHA 72h |  | -127.243 |  | -163.260 |  | -91.226 |  | 10.944 |  | -11.627 |  | < .001 |  |
|  |  | LHA 72h |  | -190.409 |  | -226.426 |  | -154.392 |  | 10.944 |  | -17.399 |  | < .001 |  |
| HCC 72h |  | HHA 72h |  | 88.160 |  | 52.143 |  | 124.177 |  | 10.944 |  | 8.056 |  | < .001 |  |
|  |  | LHA 72h |  | 24.994 |  | -11.023 |  | 61.011 |  | 10.944 |  | 2.284 |  | 0.227 |  |
| HHA 72h |  | LHA 72h |  | -63.166 |  | -99.183 |  | -27.149 |  | 10.944 |  | -5.772 |  | 0.001 |  |
|  | | | | | | | | | | | | | | | |
| *Note.*  P-value and confidence intervals adjusted for comparing a family of 5 estimates (confidence intervals corrected using the tukey method). | | | | | | | | | | | | | | | |
