# Supplementary material for: An in vitro study to assess the effect of hyaluronan-based gels on muscle-derived cells: Highlighting a new perspective in regenerative medicine
Source: PLoS One. 2020 Aug 6;15(8):e0236164. doi: 10.1371/journal.pone.0236164 (PMC7410276; doi:10.1371/journal.pone.0236164)
Supplement: S3 Fig — (DOCX) [file pone.0236164.s003.docx]

**Figure 5A**

*p-value <0.05

**p-value<0.01

***p-value<0.001

**Results**

**ANOVA**

| **ANOVA - qRT-PCR Atrogin** | | | | | | | | | | | |
| --- | --- | --- | --- | --- | --- | --- | --- | --- | --- | --- | --- |
| **Cases** | | **Sum of Squares** | | **df** | | **Mean Square** | | **F** | | **p** | |
| V1 |  | 179.023 |  | 4 |  | 44.756 |  | 290.082 |  | < .001 |  |
| Residuals |  | 1.543 |  | 10 |  | 0.154 |  |  |  |  |  |
|  | | | | | | | | | | | |
| *Note.*  Type III Sum of Squares | | | | | | | | | | | |

**Post Hoc Tests**

**Standard**

| **Post Hoc Comparisons - V1** | | | | | | | | | | | | | | | |  |
| --- | --- | --- | --- | --- | --- | --- | --- | --- | --- | --- | --- | --- | --- | --- | --- | --- |
|  | | | | | | **95% CI for Mean Difference** | | | |  | | | | | |  |
|  | |  | | **Mean Difference** | | **Lower** | | **Upper** | | **SE** | | **t** | | **p _tukey Symbol_** | |  |
| CTR |  | H-HA |  | 8.152 |  | 7.096 |  | 9.207 |  | 0.321 |  | 25.417 |  | < .001 | *** |  |
|  |  | HCC |  | 7.175 |  | 6.119 |  | 8.230 |  | 0.321 |  | 22.371 |  | < .001 | *** |  |
|  |  | L-HA |  | 0.007 |  | -1.049 |  | 1.062 |  | 0.321 |  | 0.021 |  | 1.000 |  |  |
|  |  | TNF-alpha |  | 3.024 |  | 1.969 |  | 4.080 |  | 0.321 |  | 9.430 |  | < .001 | *** |  |
| H-HA |  | HCC |  | -0.977 |  | -2.032 |  | 0.079 |  | 0.321 |  | -3.046 |  | 0.073 |  |  |
|  |  | L-HA |  | -8.145 |  | -9.200 |  | -7.089 |  | 0.321 |  | -25.396 |  | < .001 | *** |  |
|  |  | TNF-alpha |  | -5.127 |  | -6.183 |  | -4.072 |  | 0.321 |  | -15.988 |  | < .001 | *** |  |
| HCC |  | L-HA |  | -7.168 |  | -8.224 |  | -6.113 |  | 0.321 |  | -22.350 |  | < .001 | *** |  |
|  |  | TNF-alpha |  | -4.151 |  | -5.206 |  | -3.095 |  | 0.321 |  | -12.942 |  | < .001 | *** |  |
| L-HA |  | TNF-alpha |  | 3.018 |  | 1.962 |  | 4.073 |  | 0.321 |  | 9.409 |  | < .001 | *** |  |
|  | | | | | | | | | | | | | | | |  |
| *Note.*  P-value and confidence intervals adjusted for comparing a family of 5 estimates (confidence intervals corrected using the tukey method). | | | | | | | | | | | | | | | |  |

**Results**

**ANOVA**

| **ANOVA - qRT-PCR FoxO3a** | | | | | | | | | | | |
| --- | --- | --- | --- | --- | --- | --- | --- | --- | --- | --- | --- |
| **Cases** | | **Sum of Squares** | | **df** | | **Mean Square** | | **F** | | **p** | |
| V1 |  | 44.444 |  | 4 |  | 11.111 |  | 77.518 |  | < .001 |  |
| Residuals |  | 2.150 |  | 15 |  | 0.143 |  |  |  |  |  |
|  | | | | | | | | | | | |
| *Note.*  Type III Sum of Squares | | | | | | | | | | | |

**Post Hoc Tests**

**Standard**

| **Post Hoc Comparisons - V1** | | | | | | | | | | | | | | | |  |
| --- | --- | --- | --- | --- | --- | --- | --- | --- | --- | --- | --- | --- | --- | --- | --- | --- |
|  | | | | | | **95% CI for Mean Difference** | | | |  | | | | | |  |
|  | |  | | **Mean Difference** | | **Lower** | | **Upper** | | **SE** | | **t** | | **p _tukey Symbol_** | |  |
| CTR |  | HCC |  | 1.701 |  | 0.874 |  | 2.527 |  | 0.268 |  | 6.352 |  | < .001 | *** |  |
|  |  | HHA |  | 2.164 |  | 1.337 |  | 2.990 |  | 0.268 |  | 8.082 |  | < .001 | *** |  |
|  |  | LHA |  | 3.437 |  | 2.610 |  | 4.263 |  | 0.268 |  | 12.837 |  | < .001 | *** |  |
|  |  | TNF-alpha |  | -0.688 |  | -1.515 |  | 0.139 |  | 0.268 |  | -2.570 |  | 0.012 * |  |  |
| HCC |  | HHA |  | 0.463 |  | -0.364 |  | 1.290 |  | 0.268 |  | 1.730 |  | 0.447 |  |  |
|  |  | LHA |  | 1.736 |  | 0.909 |  | 2.563 |  | 0.268 |  | 6.485 |  | < .001 | *** |  |
|  |  | TNF-alpha |  | -2.389 |  | -3.215 |  | -1.562 |  | 0.268 |  | -8.922 |  | < .001 | *** |  |
| HHA |  | LHA |  | 1.273 |  | 0.446 |  | 2.100 |  | 0.268 |  | 4.755 |  | 0.002 | ** |  |
|  |  | TNF-alpha |  | -2.852 |  | -3.678 |  | -2.025 |  | 0.268 |  | -10.652 |  | < .001 | *** |  |
| LHA |  | TNF-alpha |  | -4.125 |  | -4.951 |  | -3.298 |  | 0.268 |  | -15.407 |  | < .001 | *** |  |
|  | | | | | | | | | | | | | | | |  |
| *Note.*  P-value and confidence intervals adjusted for comparing a family of 5 estimates (confidence intervals corrected using the tukey method). | | | | | | | | | | | | | | | |  |

**Results**

**ANOVA**

| **ANOVA - qRT-PCR MuRF-1** | | | | | | | | | | | |
| --- | --- | --- | --- | --- | --- | --- | --- | --- | --- | --- | --- |
| **Cases** | | **Sum of Squares** | | **df** | | **Mean Square** | | **F** | | **p** | |
| V1 |  | 83.023 |  | 4 |  | 20.756 |  | 293.959 |  | < .001 |  |
| Residuals |  | 0.706 |  | 10 |  | 0.071 |  |  |  |  |  |
|  | | | | | | | | | | | |
| *Note.*  Type III Sum of Squares | | | | | | | | | | | |

**Post Hoc Tests**

**Standard**

| **Post Hoc Comparisons - V1** | | | | | | | | | | | | | | | |  |
| --- | --- | --- | --- | --- | --- | --- | --- | --- | --- | --- | --- | --- | --- | --- | --- | --- |
|  | | | | | | **95% CI for Mean Difference** | | | |  | | | | | |  |
|  | |  | | **Mean Difference** | | **Lower** | | **Upper** | | **SE** | | **t** | | **p _tukey Symbol_** | |  |
| CTR |  | HCC |  | 1.245 |  | 0.531 |  | 1.959 |  | 0.217 |  | 5.740 |  | 0.001 | ** |  |
|  |  | HHA |  | 6.610 |  | 5.896 |  | 7.324 |  | 0.217 |  | 30.465 |  | < .001 | *** |  |
|  |  | LHA |  | 3.823 |  | 3.109 |  | 4.537 |  | 0.217 |  | 17.619 |  | < .001 | *** |  |
|  |  | TNF-alpha |  | 4.467 |  | 3.752 |  | 5.181 |  | 0.217 |  | 20.587 |  | < .001 | *** |  |
| HCC |  | HHA |  | 5.364 |  | 4.650 |  | 6.078 |  | 0.217 |  | 24.725 |  | < .001 | *** |  |
|  |  | LHA |  | 2.577 |  | 1.863 |  | 3.291 |  | 0.217 |  | 11.879 |  | < .001 | *** |  |
|  |  | TNF-alpha |  | 3.221 |  | 2.507 |  | 3.935 |  | 0.217 |  | 14.846 |  | < .001 | *** |  |
| HHA |  | LHA |  | -2.787 |  | -3.501 |  | -2.073 |  | 0.217 |  | -12.846 |  | < .001 | *** |  |
|  |  | TNF-alpha |  | -2.143 |  | -2.857 |  | -1.429 |  | 0.217 |  | -9.878 |  | < .001 | *** |  |
| LHA |  | TNF-alpha |  | 0.644 |  | -0.070 |  | 1.358 |  | 0.217 |  | 2.967 |  | 0.082 |  |  |
|  | | | | | | | | | | | | | | | |  |
| *Note.*  P-value and confidence intervals adjusted for comparing a family of 5 estimates (confidence intervals corrected using the tukey method). | | | | | | | | | | | | | | | |  |
