# Supplementary material for: An in vitro study to assess the effect of hyaluronan-based gels on muscle-derived cells: Highlighting a new perspective in regenerative medicine
Source: PLoS One. 2020 Aug 6;15(8):e0236164. doi: 10.1371/journal.pone.0236164 (PMC7410276; doi:10.1371/journal.pone.0236164)
Supplement: S4 Fig — (DOCX) [file pone.0236164.s004.docx]

**Figure 6A**

**Results**

**ANOVA**

| **ANOVA - qRT-PCR Desmin** | | | | | | | | | | | |
| --- | --- | --- | --- | --- | --- | --- | --- | --- | --- | --- | --- |
| **Cases** | | **Sum of Squares** | | **df** | | **Mean Square** | | **F** | | **p** | |
| V1 |  | 7.833 |  | 4 |  | 1.958 |  | 22.884 |  | < .001 |  |
| Residuals |  | 0.856 |  | 10 |  | 0.086 |  |  |  |  |  |
|  | | | | | | | | | | | |
| *Note.*  Type III Sum of Squares | | | | | | | | | | | |

**Post Hoc Tests**

**Standard**

| **Post Hoc Comparisons - V1** | | | | | | | | | | | | | | |  |  |
| --- | --- | --- | --- | --- | --- | --- | --- | --- | --- | --- | --- | --- | --- | --- | --- | --- |
|  | | | | | | **95% CI for Mean Difference** | | | |  | | | | | |  |
|  | |  | | **Mean Difference** | | **Lower** | | **Upper** | | **SE** | | **t** | | **p _tukey_** | |  |
| CTR |  | HCC |  | 2.047 |  | 1.261 |  | 2.833 |  | 0.239 |  | 8.570 |  | < .001 |  |  |
|  |  | HHA |  | 1.314 |  | 0.528 |  | 2.100 |  | 0.239 |  | 5.502 |  | 0.002 |  |  |
|  |  | LHA |  | 0.765 |  | -0.021 |  | 1.551 |  | 0.239 |  | 3.203 |  | 0.057 |  |  |
|  |  | TNF-alpha |  | 0.356 |  | -0.430 |  | 1.142 |  | 0.239 |  | 1.490 |  | 0.590 |  |  |
| HCC |  | HHA |  | -0.733 |  | -1.519 |  | 0.053 |  | 0.239 |  | -3.067 |  | 0.055 |  |  |
|  |  | LHA |  | -1.282 |  | -2.068 |  | -0.496 |  | 0.239 |  | -5.367 |  | 0.002 |  |  |
|  |  | TNF-alpha |  | -1.691 |  | -2.477 |  | -0.905 |  | 0.239 |  | -7.079 |  | < .001 |  |  |
| HHA |  | LHA |  | -0.549 |  | -1.335 |  | 0.237 |  | 0.239 |  | -2.299 |  | 0.222 |  |  |
|  |  | TNF-alpha |  | -0.958 |  | -1.744 |  | -0.172 |  | 0.239 |  | -4.012 |  | 0.016 |  |  |
| LHA |  | TNF-alpha |  | -0.409 |  | -1.195 |  | 0.377 |  | 0.239 |  | -1.713 |  | 0.468 |  |  |
|  | | | | | | | | | | | | | | | |  |
| *Note.*  P-value and confidence intervals adjusted for comparing a family of 5 estimates (confidence intervals corrected using the tukey method). | | | | | | | | | | | | | | | |  |

**Results**

**ANOVA**

| **ANOVA - qRT-PCR myogenin** | | | | | | | | | | | |
| --- | --- | --- | --- | --- | --- | --- | --- | --- | --- | --- | --- |
| **Cases** | | **Sum of Squares** | | **df** | | **Mean Square** | | **F** | | **p** | |
| V1 |  | 46.787 |  | 4 |  | 11.697 |  | 41.133 |  | < .001 |  |
| Residuals |  | 2.844 |  | 10 |  | 0.284 |  |  |  |  |  |
|  | | | | | | | | | | | |
| *Note.*  Type III Sum of Squares | | | | | | | | | | | |

**Post Hoc Tests**

**Standard**

| **Post Hoc Comparisons - V1** | | | | | | | | | | | | | | | |  |
| --- | --- | --- | --- | --- | --- | --- | --- | --- | --- | --- | --- | --- | --- | --- | --- | --- |
|  | | | | | | **95% CI for Mean Difference** | | | |  | | | | | |  |
|  | |  | | **Mean Difference** | | **Lower** | | **Upper** | | **SE** | | **t** | | **p _tukey_** | |  |
| CTR |  | HCC |  | 1.986 |  | 0.553 |  | 3.419 |  | 0.435 |  | 4.561 |  | 0.007 |  |  |
|  |  | HHA |  | 1.253 |  | -0.180 |  | 2.686 |  | 0.435 |  | 2.878 |  | 0.095 |  |  |
|  |  | LHA |  | 0.704 |  | -0.729 |  | 2.137 |  | 0.435 |  | 1.617 |  | 0.520 |  |  |
|  |  | TNF-alpha |  | -3.118 |  | -4.551 |  | -1.685 |  | 0.435 |  | -7.162 |  | < .001 |  |  |
| HCC |  | HHA |  | -0.733 |  | -2.166 |  | 0.700 |  | 0.435 |  | -1.683 |  | 0.484 |  |  |
|  |  | LHA |  | -1.282 |  | -2.715 |  | 0.151 |  | 0.435 |  | -2.944 |  | 0.045 |  |  |
|  |  | TNF-alpha |  | -5.104 |  | -6.537 |  | -3.671 |  | 0.435 |  | -11.722 |  | < .001 |  |  |
| HHA |  | LHA |  | -0.549 |  | -1.982 |  | 0.884 |  | 0.435 |  | -1.261 |  | 0.719 |  |  |
|  |  | TNF-alpha |  | -4.371 |  | -5.804 |  | -2.938 |  | 0.435 |  | -10.040 |  | 0.048 |  |  |
| LHA |  | TNF-alpha |  | -3.822 |  | -5.255 |  | -2.389 |  | 0.435 |  | -8.778 |  | 0 .081 |  |  |
|  | | | | | | | | | | | | | | | |  |
| *Note.*  P-value and confidence intervals adjusted for comparing a family of 5 estimates (confidence intervals corrected using the tukey method). | | | | | | | | | | | | | | | |  |
