# Supplementary material for: An in vitro study to assess the effect of hyaluronan-based gels on muscle-derived cells: Highlighting a new perspective in regenerative medicine
Source: PLoS One. 2020 Aug 6;15(8):e0236164. doi: 10.1371/journal.pone.0236164 (PMC7410276; doi:10.1371/journal.pone.0236164)
Supplement: S1 Raw images — (PDF) [file pone.0236164.s005.pdf]

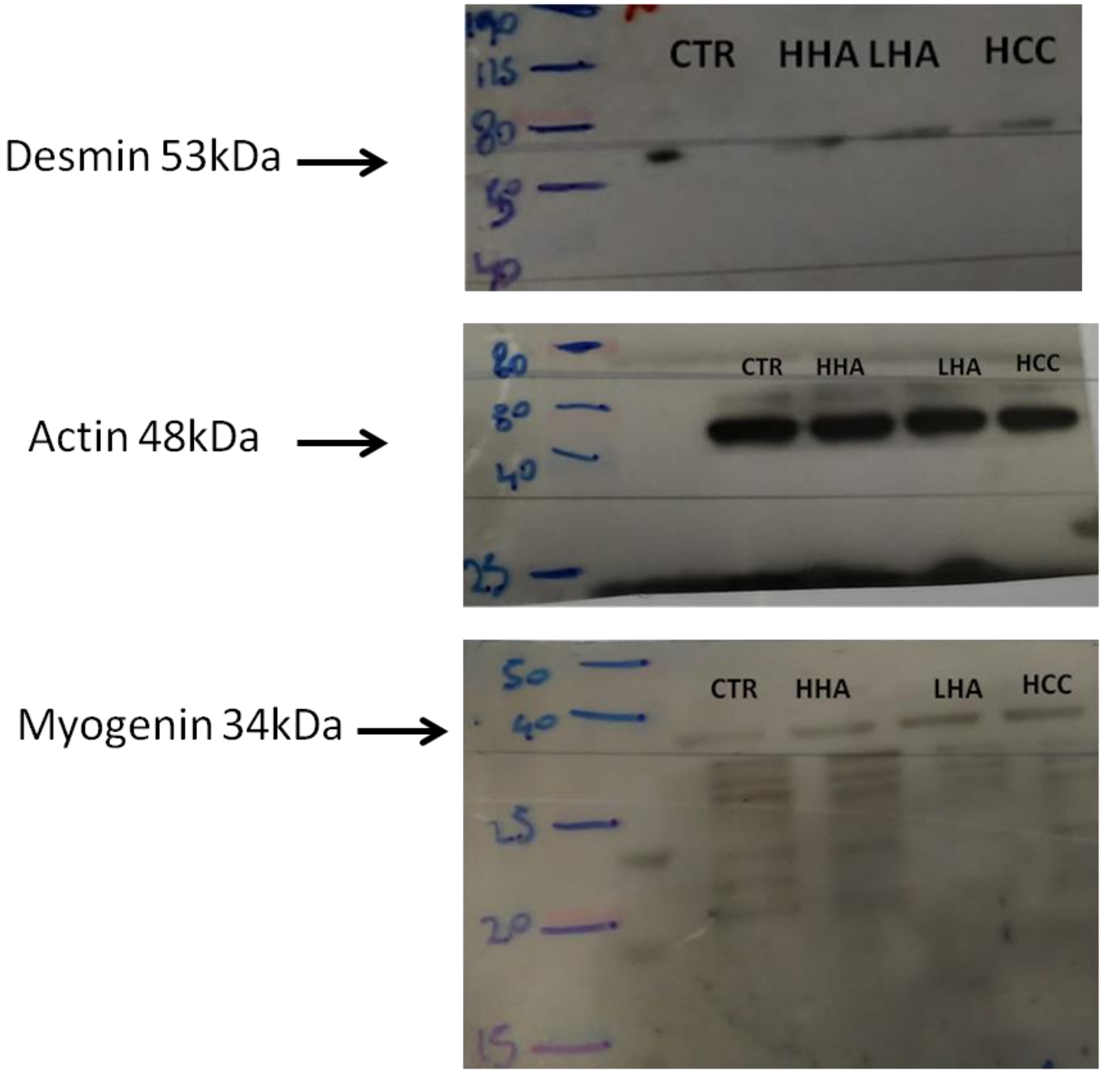
Desmin 53kDa
CTR
HHA
LHA
HCC
Actin 48kDa
CTR
HHA
LHA
HCC
Myogenin 34kDa
CTR
HHA
LHA
HCC

Fig2C

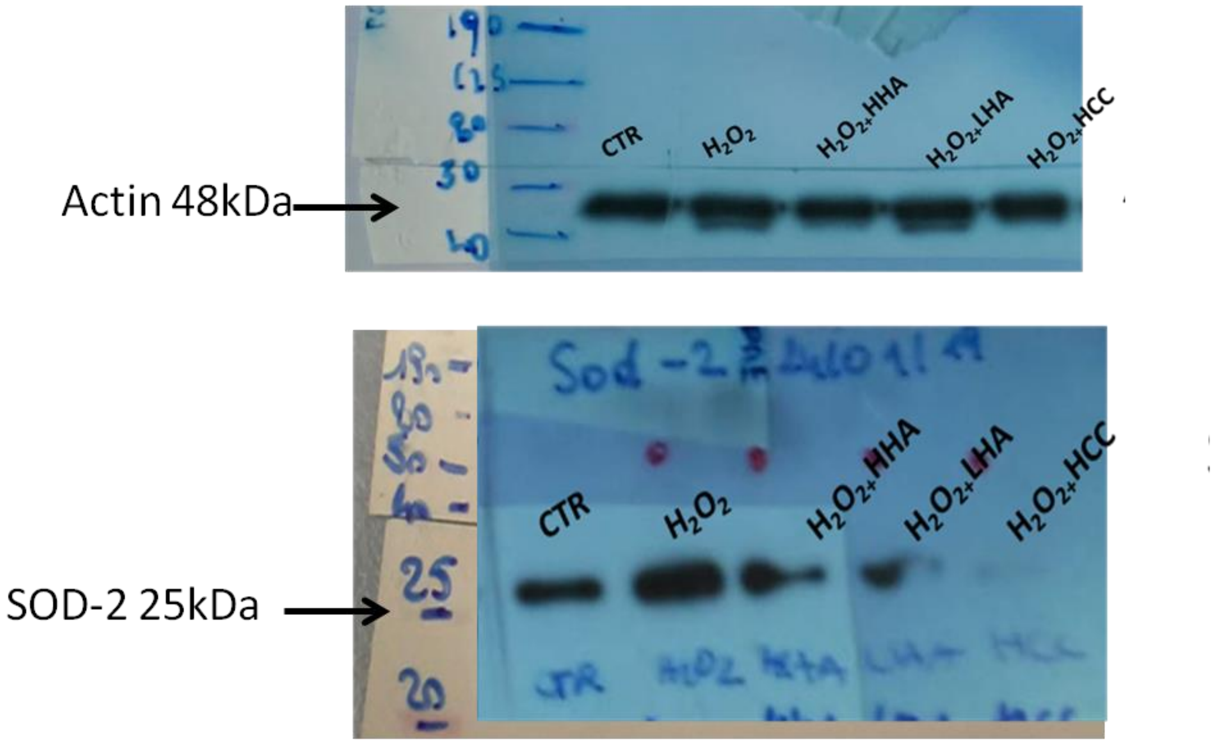
Actin 48kDa
CTR
H_2O_2
H_2O_2+HHA
H_2O_2+LHA
H_2O_2+HCC
SOD-2 25kDa
CTR
H_2O_2
H_2O_2+HHA
H_2O_2+LHA
H_2O_2+HCC

Fig4A

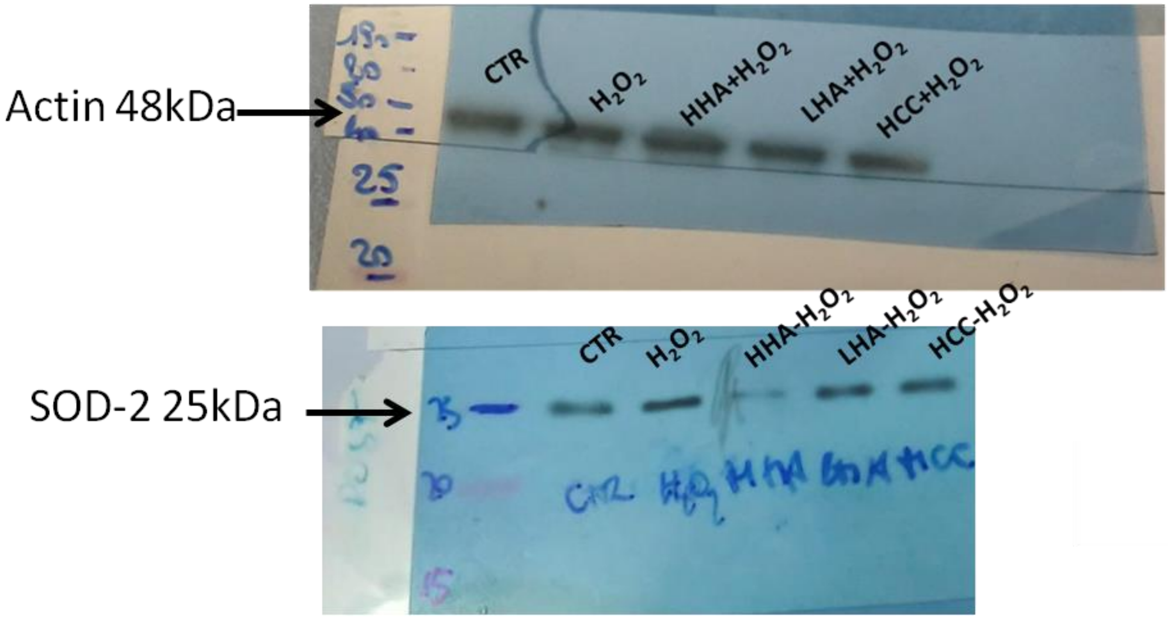
Actin 48kDa
CTR
H_2O_2
HHA+H_2O_2
LHA+H_2O_2
HCC+H_2O_2
SOD-2 25kDa
CTR
H_2O_2
HHA-H_2O_2
LHA-H_2O_2
HCC-H_2O_2

Fig4B

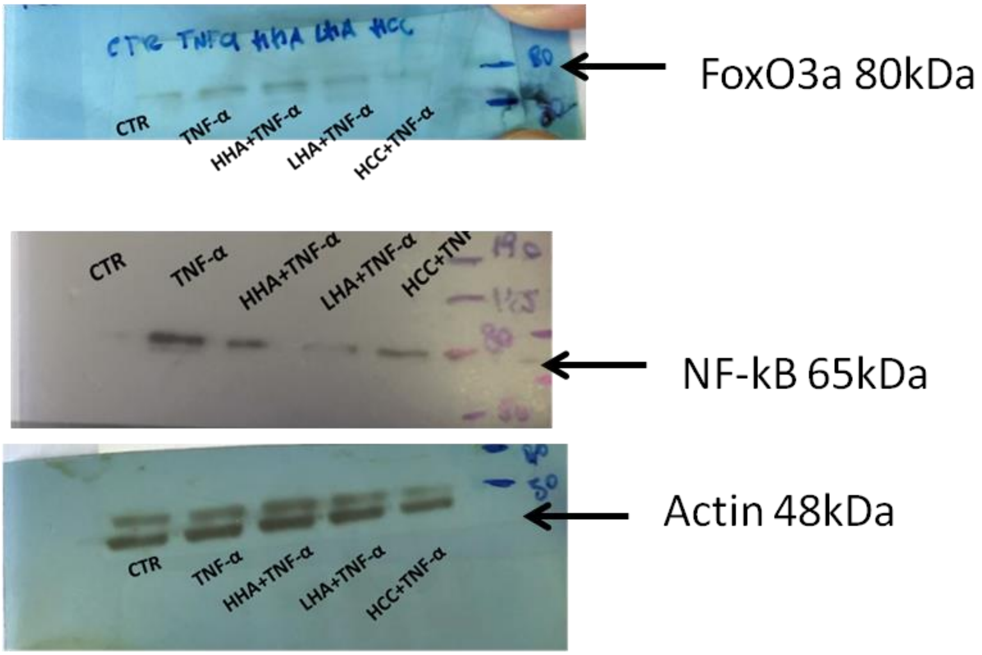
FoxO3a 80kDa
CTR
TNF-α
HHA+TNF-α
LHA+TNF-α
HCC+TNF-α
NF-kB 65kDa
Actin 48kDa

Fig5B

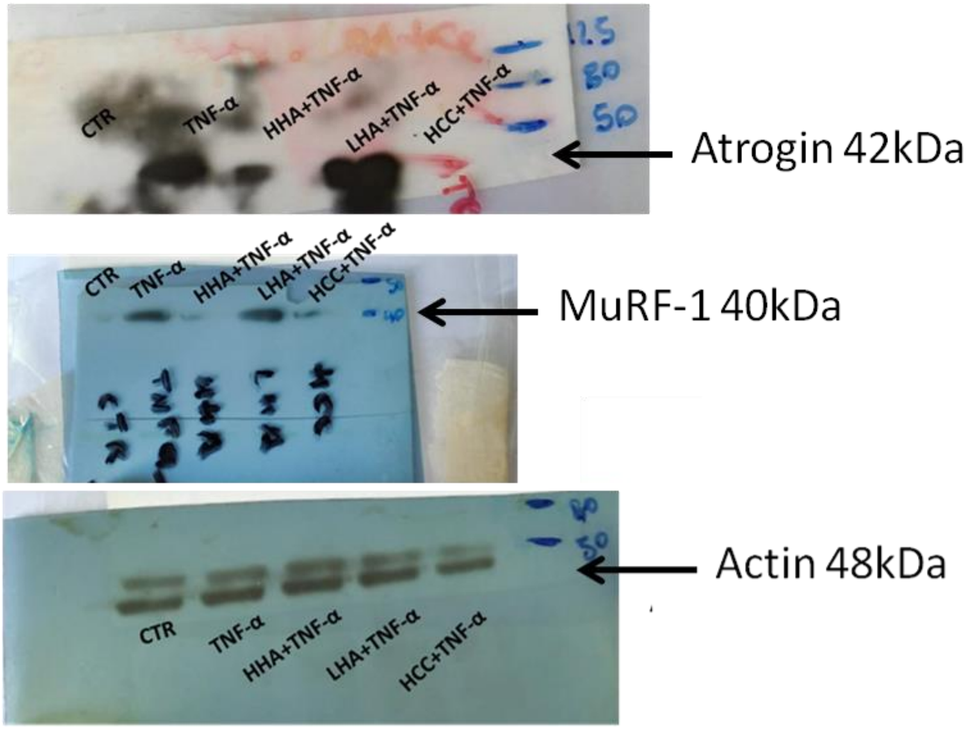
Atrogin 42kDa
CTR
TNF-α
HHA+TNF-α
LHA+TNF-α
HCC+TNF-α
MuRF-1 40kDa
Actin 48kDa

Fig5C

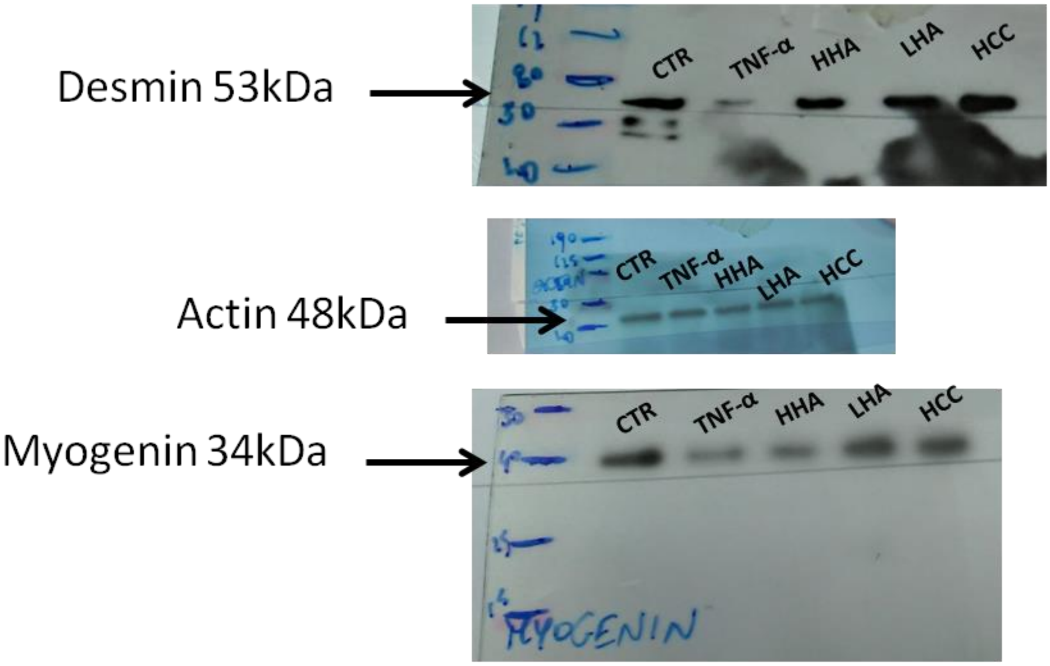
Desmin 53kDa
CTR
TNF-α
HHA
LHA
HCC
Actin 48kDa
CTR
TNF-α
HHA
LHA
HCC
Myogenin 34kDa
CTR
TNF-α
HHA
LHA
HCC
MYOGENIN

Fig6B
