# Supplementary material for: An in vitro study to assess the effect of hyaluronan-based gels on muscle-derived cells: Highlighting a new perspective in regenerative medicine
Source: PLoS One. 2020 Aug 6;15(8):e0236164. doi: 10.1371/journal.pone.0236164 (PMC7410276; doi:10.1371/journal.pone.0236164)
Supplement: Attachment — Submitted filename: Response to Reviewers PONE-D-20-03354.docx [file pone.0236164.s006.docx]

Dear Editor,

Thank you for your consideration for our manuscript.

Hereby we submit the revised version of the manuscript number PONE-D-20-03354

“An in vitro study to assess the effect of hyaluronan-based gels on muscle-derived cells: Highlighting a new perspective in regenerative medicine” PLOS ONE

We considered the reviewer suggestions and the manuscript was modified and corrected accordingly.

All the minor points were addressed and modified in the text, the manuscript with track changes revisions (revised manuscript with track changes) is provided beside the “clean”version (“manuscript” without tracked changes) according to the PlosOne author guidelines.

The original uncropped and unadjusted images underlying all blot results are reported in Supporting information files.

Please find following the response to reviewers:

Response to Reviewer #1:

5. Review Comments to the Author

Please use the space provided to explain your answers to the questions above. You may also include additional comments for the author, including concerns about dual publication, research ethics, or publication ethics. (Please upload your review as an attachment if it exceeds 20,000 characters)

Reviewer #1: The authors describe the effects of HA of high and low mol. mass and conjugated HA on rat muscle derived cells. Authors studied cell proliferation, viability and in vitro atrophy finding that conjugated HA had the better effects.

The manuscript is clearly written and could be of interest in several clinical applications.

Several points, however, need to be addressed by the authors to increase discuss and to hypothesize the

molecular mechanisms that trigger such HA treatments.

Have the authors an hypothesis of the mechanism of HCC? could HCC uses similar receptors of LHA and HHA?

HCC is based on the cooperativity of hydrogen bonding among che high molecular weight Hyaluronan chains and the low molecular weight ones. These are thermally stabilized. However, when diluted in the cell media, or in physiological environment the competition of solvent is becoming more and more effective in time releasing both the low and high molecular weight chains. For this reason, it is possibly the direct interaction of the slowly released single chains to release the effect. A study focus on the direct interaction of the complex on the cell wall specific receptors was not carried out yet. However, we think that the suggestion of the editor/referee is very interesting starting point for in depth analyses of the unraveled features of these HCC.

Are the different HA added stable in the cell medium or such HA are degraded (a measurement of HYALs could be usefull to discuss this point)?

HCC are more stable than HHA to hyaluronidases, specifically 80% of the initial molecular population with MW higher than 1 MDa was digested in 24h in presence of hyaluronidase (BTH 0.5 U/mL at 37°C) while 5% only was degraded of the same fraction in HCC (D’Agostino et al. 2015). Very few studies on the hyaluronidases synthesis *in vitro* are presented in literature, however the most recent is addressing this point regarding mast cells (Calve et al. [J Biol Chem.](https://www.ncbi.nlm.nih.gov/pubmed/31175155) 2019 Jul 26;294(30):11458-11472). In previous research paper we evaluated in a similar time scale the effect of HCC compared to HHA and LHA and generally the medium (containing the gel preparation) was exchanged every 48h.

Many function of HA are know to be mediated by HC-HA complexes formed by TSG6. Have the authors

investigated whether or not TSG6 could be involved?

TSG6 has been demonstrated to interact with HA (Spinelli et al., Milner et al., and Baranova et al.).

Caroline and collaborators propose a high expression level in skeletal muscle for this reason we may suppose that among the molecular mechanism (some of which mainly related to CD44) responsible for the biological effect also TSG6 may be involved. In this respect it will be of great interest a specific assessment of the HCC interaction in comparison to the sole high and low molecular weight fraction, not only in the muscle cell line, but also in other in vitro model (e.g. OA). The authors have been discussed potential function of TSG-6 referring our study in the discussion section (page 17 line 607-615).

As HCC is patented by one of the authors, is there any conflict of interest?

Prof Schiraldi is among the inventors, but the assignee is a company, since the research work was developed within a public-private joint project partially financed by Campania regional government (measure for innovation). We may add a specification in the section conflict of interest to specify this.

Response to Reviewer #2:

Reviewer #2: The manuscript demonstrates that a composite of high and low molecular weight hyaluronan (HCC) better preserves muscle cell phenotype than high molecular weight hyaluronan (HHA) or low molecular weight hyaluronan (LHA) in preserving phenotype of primary muscle cells and protect them from atrophy. While this finding is interesting, there are notable details lacking from the methods (e.g., description of controls) and some questions about the data that make it difficult to interpret. Details are provided in the comments below.

1. Please add an “and” before “c)” in the 3rd sentence to complete this list.

We added “and” before “c)” in the 3rd sentence to complete the list (page 8 line 163).

2. The abstract states: “The results showed that HCC and HHA increased cell proliferation by 1.15 and 2.3 folds in comparison to control, respectively.” Please clarify what the control is here in the text. It is also not clear from the Methods or Results text what the control conditions are.

In this sentence (page 2 line 38) we replaced “control” with “un-treated cells”. Also, we clarified the description of all the control conditions used for the three experimental set-up investigated from the Methods and Results text.

3. The abstract states: “In this model, HCC revealed a noteworthy beneficial effect on the myogenic biomarkers indicating that it could be used as a promising platform for tissue regeneration with specific attention to muscle cell protection against stressful agents.” However, text up to this point indicates that HHA worked at least as well, if not better, than HCC.

Yes, this is the meaning we wanted to give (page 2 line 40).

However, in figure 5 A, B and C a significant better effect from HCC was found in reducing atrogens expression. Results relative to oxidative stress (Fig 3A) and atrophy (fig 5C) HCC and HHA presented similar behavior counteracting stressful conditions. We underline that HHA resulted slightly more effective than HCC in reducing SOD-2 expression during protective effect (fig4B).

4. Introduction, pg. 4: “HA is a hygroscopic molecule that is able to synthesize the ECM…” The use of “synthesize” is not appropriate here. The HA does not produce the ECM, although it can act to organize it structurally by complexing with other ECM macromolecules.

We apologize, the sentence was not correct, we modified that inserting:

“HA is a hygroscopic molecule that is able to structurally organize the ECM by complexing with other ECM macromolecules”, page 4 line 78-79 in the introduction section.

5. Method, Pg. 8, paragraph 3- Please provide more information on the fluorescence microscope such as what brand and model was used.

Immufluorescence analyses was accomplished using an Axiovert 200 (Zeiss) microscope, detail have been added to the materials and methods section page 9 line 189-190.

6. Method, pg.9, paragraph 2- Authors state that “Cytotoxicity was assessed using 3.0 × 104 cells seeded in a standard 24-well culture plate, pre- treated with 50 μM H_2_O_2_ (30 min), and then treated with HHA, LHA, and HCC, respectively (0.16% w/v) for 24 h. Analyses were performed after 24, 48, and 96 h post-treatment by measuring the reduction of the tetrazolium dye 3-(4,5-dimethylthiazol-2-yl)-2,5-diphenyltetrazolium bromide (MTT) “. Does this mean that HA was kept in the medium for only the first 24 hrs? Later, in the Results on pg. 12, the text implies that instead the times of HA treatments were different. Please clarify this issue.

Thanks for the comment, we have clarified the description of the procedure performed in the methods section page 9 line 207 and modified the outcomes considering the metabolic activity in the results section line starting from page 13 line 283-298 (fig 3A and B).

7. How was the concentration of HA controlled for because HHA, LHA and HCC experimental conditions?

Respect to the initial gels, that were prepared from pharma grade powder at 16 g/L and thermally treated for 12 min at 120°C in the autoclave, we diluted them into the cell medium 1:10 to achieve a final concentration of 1.6mg/mL. For HCC we used a sterile syringes from IBSA Italia (IBSA Farmaceutici Italia Srl, Italy) diluted 1:20 into the cell medium, as reported in materials and methods section page 5 line 115.

8. Were different concentrations of HA in each solution evaluated? It will be important to know if these effects are concentration dependent.

No, we have used only one concentration (1.6 mg/mL) for all hyaluronans tested.

9. Please add indications onto graphs in Figures to show where data are significantly different from each other. This has only been done in Figures 3 and 6. It would also help to state when findings are statistically significant when discussing them in the Results text.

As requested by the referee we have performed t- student test analyses on all figures as suggested, and modified accordingly the figures and texts.

10. Figure 1 is never referred to in the text.

We have mentioned the figure 1 referring to atrophy signaling at page 3 line 59 of the introduction section.

11. Table 1, pg.10.- Table does not look very polished: e.g. some Genbank accession no are underlined while others are not, some rows are wider than others, etc.

According to the suggestion the table 1 was formatted according to plos one guidelines and inserted in the manuscript.

12. Results: The subtitles like “(experimental Set-up a)” are not really necessary.

We deleted subtitles as suggested by the referee.

13. This may have happened during pdf conversion, but all of the figures have blurry lines and text.

We have upload figures as a 300 dpi following the journal guidelines however, we will up-load those again hoping that their quality will be improved.

14. In Figure 2B, the images require scale bars.

We apologize, on the original figure scale bars were present and visible. The issue may be due to the pdf conversion. We will try to re-upload the image to make it better visible.

15. The procedures for hydrogen peroxide “pre-challenge” and “post-challenge” are not clear from the methods or figure 4 caption.

Ok, we have modified the description for oxidative stress in the materials and methods page 7 from line 150-158.

We aimed at evaluating if the hyaluronan gel were able to:

1) counteract the detrimental effect of Hydrogen peroxide treatment- and in this case the cells were initially challenged with H_2_O_2_ and successively treated with the gels.
2) To prevent the damage of stressfull agent- and in this case the cells were initially treated with the gels and then exposed (challenged) with H_2_O_2_.

We called case A) Rescue (repair function); while case B is a prevention/alert or protective function of the gels.

16. As the MTT assay does not explicitly measure numbers of viable cells, it is more appropriate to refer to MTT results as reflective of the cells’ metabolic activity.

We agree with the referee that the figure axis may be misleading to the readers for this reason we modified the text indicating MTT results as cells’ metabolic activity page 13 line 283-298.

17. Statistical analyses: Students t-tests are not appropriate for these data sets where multiple comparisons are being made or where multiple independent variables are evaluated (e.g., Fig. 3, where time and treatment are variables). Please also include the software used for statistical analysis in the Methods.

We have used Microsoft excel in order to perform t-student test analyses. In particular, we compared the efficacy of different HAs treatments in counteracting H_2_O_2_ effect. For all time investigated (24, 48 and 72h) all HAs tested were effective respect to H_2_O_2_ treatment. However, the effect of HCC is more noticeable at 72h in the rescue/repair function (fig3A). Also, the HAs treatments resulted significant (p<0.01) with respect to H_2_O_2_ treatment at 48 and 72h in the protection function condition (Fig.3B).

18. Results: For all graphs, it is not clear what the asterisks indicating significance are comparing to here. For example, in Fig. 5A, is HHA MuRF-1 different than HHA FoxO3a? For LHA and HCC, do the different genes have different expression than the other genes or are all genes different than TNF-α?

For figure 5A statistical analyses (t-test *p<0.01) were performed for each gene of different HAs treatment respect to TNF-α insult. Our purpose was to demonstrate that TNF-α insult induces atrogenes up-regulation and HAs treatment was effective in reducing it. HAs counteract the detrimental effect of TNF-α that is recognized as a treatment resembling atrophic conditions (Cho et al. 2018 ref 28 in the present manuscript).

19. Results: For all figures, please indicate what the error bars represent (e.g., Standard deviation/ SEM?) in the captions.

As requested, we indicated the error bars as standard deviation in the figure captions.

20. Results: For all figures, please indicate what the number of replicates used for data analysis in the captions.

We indicated the number of replicates used for our data analyses in the figure captions.

21. Results, Fig 3: Was there a positive control for this experiment?

The positive control (of stress) was the H_2_O_2_ treated cells while the negative control was the untreated-cells.

Untreated cells are the control

Stressed cells are the detrimental conditions.

On the already stressed cells were run to evaluate the biological effect of HAs.

22. Result, Fig 4: What are the negative and positive controls for this experiment?

The positive control (of stress) was the H_2_O_2_ treated cells while the negative control was the untreated-cells.

Untreated cells are the control

Stressed cells are the detrimental conditions.

On the already stressed cells were run to evaluate the biological effect of HAs.

23. Results, Fig 5. (B, C): The actin control rows appear to be identical in both western blot figures. Were these all run on the same blot? Also, the HCC treatment condition appears to have more actin. Is there a reason for this?

It is in fact a single western blot where we have separated the genes according to their function. For all experiment we have loaded the same protein amount (10μg tot) for all biomarkers investigated and also for actin. In this respect, actin signal is used as housekeeping protein as internal control for normalization.

24. Results, Fig 6(B). For western blotting sample, random hyphens were added to the LHA and HHA the

abbreviations.

Thank you for the remark, a mistake was made. We changed the name of samples (in the results section fig 6.B).

25. Discussion, pg. 17: Please be consistent in italic style of “in vitro”.

We controlled the italics for “in vitro” throughout the document.

26. Supporting information, S1B: Background in the red channel seems pretty high.

Ok, we have re-acquired the image by lowering the phalloidin red channel, for figure S1B.

We thank the reviewers for their suggestions that greatly improved our manuscript.

Kind regards

Chiara Schiraldi

Antonietta Stellavato
