# Supplementary material for: An in vitro study to assess the effect of hyaluronan-based gels on muscle-derived cells: Highlighting a new perspective in regenerative medicine
Source: PLoS One. 2020 Aug 6;15(8):e0236164. doi: 10.1371/journal.pone.0236164 (PMC7410276; doi:10.1371/journal.pone.0236164)
Supplement: Attachment — Submitted filename: Response to reviewers 16 06 2020_rev CS.docx [file pone.0236164.s007.docx]

Dear Editor,

Thank you for your consideration for our manuscript.

Hereby we submit the second revision of the manuscript number PONE-D-20-03354R1

“An in vitro study to assess the effect of hyaluronan-based gels on muscle-derived cells: Highlighting a new perspective in regenerative medicine” PLOS ONE

We considered the suggestions of reviewer 2, the manuscript was modified and corrected accordingly.

All the minor points were addressed and modified in the text, the manuscript with track changes revisions (revised manuscript with track changes) is provided beside the “clean”version (“manuscript” without tracked changes) according to the PlosOne author guidelines.

One-way ANOVA and Tukey post hoc test were performed by JASP soft-ware (https://jasp-stats.org) and are reported in Supporting information files.

Additional Editor Comments (if provided):

The manuscript has been carefully revised and improved. There is a issue related to use of statistical method T instead ANOVA. It may be addressed to confirm data robustness.


[Note: HTML markup is below. Please do not edit.]

Reviewers' comments:

Reviewer's Responses to Questions

**Comments to the Author**

1. If the authors have adequately addressed your comments raised in a previous round of review and you feel that this manuscript is now acceptable for publication, you may indicate that here to bypass the “Comments to the Author” section, enter your conflict of interest statement in the “Confidential to Editor” section, and submit your "Accept" recommendation.

Reviewer #1: All comments have been addressed

Reviewer #2: (No Response)

2. Is the manuscript technically sound, and do the data support the conclusions?

The manuscript must describe a technically sound piece of scientific research with data that supports the conclusions. Experiments must have been conducted rigorously, with appropriate controls, replication, and sample sizes. The conclusions must be drawn appropriately based on the data presented.

Reviewer #1: (No Response)

Reviewer #2: Yes

3. Has the statistical analysis been performed appropriately and rigorously?

Reviewer #1: (No Response)

Reviewer #2: No

4. Have the authors made all data underlying the findings in their manuscript fully available?

The [PLOS Data policy](http://www.plosone.org/static/policies.action#sharing) requires authors to make all data underlying the findings described in their manuscript fully available without restriction, with rare exception (please refer to the Data Availability Statement in the manuscript PDF file). The data should be provided as part of the manuscript or its supporting information, or deposited to a public repository. For example, in addition to summary statistics, the data points behind means, medians and variance measures should be available. If there are restrictions on publicly sharing data—e.g. participant privacy or use of data from a third party—those must be specified.

Reviewer #1: (No Response)

Reviewer #2: Yes

5. Is the manuscript presented in an intelligible fashion and written in standard English?

PLOS ONE does not copyedit accepted manuscripts, so the language in submitted articles must be clear, correct, and unambiguous. Any typographical or grammatical errors should be corrected at revision, so please note any specific errors here.

Reviewer #1: (No Response)

Reviewer #2: Yes

6. Review Comments to the Author

Please use the space provided to explain your answers to the questions above. You may also include additional comments for the author, including concerns about dual publication, research ethics, or publication ethics. (Please upload your review as an attachment if it exceeds 20,000 characters)

Reviewer #1: (No Response)

Reviewer #2: Thank you for your careful consideration of the previous review comments. There are still issues with the use of t-tests for statistical analysis. In all data sets, there are multiple levels of the independent variables, and thus multiple comparisons. Use of t-tests repeatedly compounds error and may give false positives. Thus, something like a one-way ANOVA (for normally distributed data) is required. T-tests are particularly not appropriate when multiple independent variables are evaluated (e.g., Fig. 3, where time and treatment are variables). Please address this issue.

We have included for Fig 3, Fig 5A and Fig 6A, the one-way ANOVA and Tukey post hoc test by JASP soft-ware (<https://jasp-stats.org>). In fact in this way we efficiently compared the different treatments and data sets. On the figures we inserted the symbols corresponding to p values for the diverse comparisons. All the data are reported as tables of the software outcomes and included as Supporting information.

Please find following the specific response to each point raised by reviewer and inserted in the manuscript accordingly:

Line 247-250 page 11 in the statistical analyses paragraph.

Line 298, 303, 305, 308 page 13 in the MMT-test results referring to Fig3.

Line 329-331 page 14 in the fig3 legend.

Line 366,368,371 page 15 for qRT-PCR results and 386-393 for fig5A legend.

Line 408 page 17 qRT-PCR results and 413-421 page 17 for fig6A legend.

We think that this further in depth analyses of experimental data set is improving the manuscript quality and for this reason we wish to thank the reviewer for the suggestion.

7. PLOS authors have the option to publish the peer review history of their article ([what does this mean?](https://journals.plos.org/plosone/s/editorial-and-peer-review-process#loc-peer-review-history)). If published, this will include your full peer review and any attached files.


If you choose “no”, your identity will remain anonymous but your review may still be made public.


**Do you want your identity to be public for this peer review?** For information about this choice, including consent withdrawal, please see our [Privacy Policy](https://www.plos.org/privacy-policy).

Reviewer #1: No

Reviewer #2: No


[NOTE: If reviewer comments were submitted as an attachment file, they will be attached to this email and accessible via the submission site. Please log into your account, locate the manuscript record, and check for the action link "View Attachments". If this link does not appear, there are no attachment files.]

While revising your submission, please upload your figure files to the Preflight Analysis and Conversion Engine (PACE) digital diagnostic tool, <https://pacev2.apexcovantage.com/>. PACE helps ensure that figures meet PLOS requirements. To use PACE, you must first register as a user. Registration is free. Then, login and navigate to the UPLOAD tab, where you will find detailed instructions on how to use the tool. If you encounter any issues or have any questions when using PACE, please email PLOS at [figures@plos.org](mailto:figures@plos.org). Please note that Supporting Information files do not need this step.
